# The validity of a behavioural multiple-mini-interview within an assessment centre for selection into specialty training

**DOI:** 10.1186/1472-6920-14-169

**Published:** 2014-08-13

**Authors:** Chris Roberts, Tyler Clark, Annette Burgess, Michael Frommer, Marcia Grant, Karyn Mossman

**Affiliations:** 1 Sydney Medical School, University of Sydney, Sydney, NSW 2006, Australia; 2 General Practice Education and Training, 10 Rudd Street, GPO Box 2914, Canberra, ACT 2601, Australia

**Keywords:** Selection, Validity, Postgraduate training, Assessment, Multiple-mini-interview, Situational judgment test, Assessment centre, General practice

## Abstract

**Background:**

Entry into specialty training was determined by a National Assessment Centre (NAC) approach using a combination of a behavioural Multiple-Mini-Interview (MMI) and a written Situational Judgement Test (SJT). We wanted to know if interviewers could make reliable and valid decisions about the non-cognitive characteristics of candidates with the purpose of selecting them into general practice specialty training using the MMI. Second, we explored the concurrent validity of the MMI with the SJT.

**Methods:**

A variance components analysis estimated the reliability and sources of measurement error. Further modelling estimated the optimal configurations for future MMI iterations. We calculated the relationship of the MMI with the SJT.

**Results:**

Data were available from 1382 candidates, 254 interviewers, six MMI questions, five alternate forms of a 50-item SJT, and 11 assessment centres. For a single MMI question and one assessor, 28% of the variance between scores was due to candidate-to-candidate variation. Interviewer subjectivity, in particular the varying views that interviewer had for particular candidates accounted for 40% of the variance in scores. The generalisability co-efficient for a six question MMI was 0.7; to achieve 0.8 would require ten questions. A disattenuated correlation with the SJT (r = 0.35), and in particular a raw score correlation with the subdomain related to clinical knowledge (r = 0.25) demonstrated evidence for construct and concurrent validity. Less than two per cent of candidates would have failed the MMI.

**Conclusion:**

The MMI is a moderately reliable method of assessment in the context of a National Assessment Centre approach. The largest source of error relates to aspects of interviewer subjectivity, suggesting enhanced interviewer training would be beneficial. MMIs need to be sufficiently long for precise comparison for ranking purposes. In order to justify long term sustainable use of the MMI in a postgraduate assessment centre approach, more theoretical work is required to understand how written and performance based test of non-cognitive attributes can be combined, in a way that achieves acceptable generalizability, and has validity.

## Background

Internationally, specialist training programs aim to produce doctors who are capable of high quality, safe, independent practice [1]. Selection procedures aspire to predict and select applicants who will go on and become able doctors, and reject those who are likely to perform poorly in future practice due to issues of professional behaviour as well as lack of clinical knowledge and skills [2]. Selection procedures involve a minimum standard of competence acceptable to a range of interested stakeholders including; employers, universities, government, the professional colleges, and the wider community. A key goal of postgraduate selection is to predict trainability prior to commencement i.e. which individuals will successfully complete training [3]. Typically, admission committees develop a ranking list of candidates, which descends in merit order until allocable places are exhausted. Traditionally postgraduate selection procedures have relied on locally derived criteria using panel interviews often supported by personal statements [4]. Over the previous decade, there have been a number of exemplars internationally where specialty colleges or government agencies have developed more robust and defensible selection procedures to determine trainability of junior doctors. Such assessments have used a wide range of formats, both written and observed, including situational judgment tests (SJT) [5], clinical problem solving test (CPST) [6], both low and high fidelity simulations [7], and the multiple-mini-interview (MMI) [8,9]. The determination of which combination of formats gives the best predictability of trainability is the subject of ongoing investigation [5,7,8]. The term assessment centre [6] is used in the selection literature to refer to a model, where candidates are required to attend a venue to undertake more than one assessment for the purpose of selection. In the postgraduate setting, there is little quantitative data on the relationship between the MMI and other assessment centre formats such as the SJT, clinical problem solving tests or simulation exercises. Similarly there has been little consideration of whether the MMI is tapping into similar or different constructs as other formats. An opportunity to investigate the reliability and the concurrent validity of the MMI compared with the SJT within a high stakes setting was provided when a national body responsible for General Practice (GP) training implemented a national assessment centre approach, in which they used both the MMI and the SJT [1]. We briefly summarise the evidence for the different kinds of validity of both assessment formats before describing the research context.

### Multiple-mini-interviews

Multiple mini-interviews have been used to assess non-cognitive characteristics of entry-level students and latterly postgraduate trainees, and were an assessment innovation based on the Objective Structured Clinical Examination (OSCE) format [10]. So far, the bulk of the evidence underpinning the MMI’s utility is from undergraduate and graduate-entry settings. Many medical schools internationally, now include the MMI as part of their selection process, for example in Canada [10,11], Australia [12,13], Saudi Arabia [14], and Israel [15]. The MMI format shows greater reliability and content validity for medical school admission processes than the traditional interviews [10,12,16-19] and is more cost-effective [20]. The reliability of the MMI in this context is moderately high, ranging from 0.65 to 0.81, most commonly being 0.7 with 8–10 questions [10,13,17,21,22]. There remains controversy as to what construct the MMI is measuring (e.g. communication, reasoning skills, entry level professionalism, non-cognitive skills, etc.) and how it relates to a network of other constructs within the assessment literature [23]. For student MMIs, there is evidence of predictive validity through moderate correlations with later clinical assessments such as the Objective Structured Clinical Examination (OSCE) [9,24]. The MMI has been used in postgraduate training selection in the United Kingdom (UK) [25], and Canada [8,9], before being introduced in Australia [1]. Early findings suggest that the MMI is a reliable and valid format for selecting junior doctors into specialty training, both for local and international medical graduates. In Canada, the generalizability coefficient of a seven station MMI for selecting applicants (n = 484 over two years) into paediatrics, obstetrics and gynaecology, and internal medicine ranged from 0.55 to 0.72, requiring 10 stations to increase reliability to 0.64-0.79 [8]. There are currently no published predictive validity studies of MMIs, which have been administered as part of postgraduate selection, although there are a number of studies under way which have yet to report.

### Situational judgement tests

SJTs are a written assessment format used to test candidates’ non-cognitive characteristics in a wide range of selection settings including health. They involve authentic, hypothetical scenarios requiring the individual to identify the most appropriate response or to rank the responses in the order they feel is most effective [7]. Evidence supporting the validity and reliability of the SJT as a shortlisting tool in postgraduate selection has prompted their introduction into the selection processes of several medical specialties within the UK. SJTs are currently an integral part of selection into general practice specialty training in the UK. Candidates are shortlisted for general practice using their scores on two written tests, an SJT and a test of cognitive skills, the clinical problem-solving test (CPST) [6]. Reliability for the SJT is typically reported as a Cronbach’s alpha of internal consistency and ranges from 0.80 - 0.83 for a 50-item test [5,26]. Criterion-related validity and predictive validity of the SJT have been demonstrated, [5] for example the SJT is a better predictor of simulated clinical performance scores than cognitive tests such as the CPST [6]. There is some information on the relationship between the SJT and the interview process. An SJT used in selection for postgraduate training in the UK (n = 2,265) showed a modest criterion-related validity (*r* = 0.52) between applicant scores on the SJT and those from a structured interview [5]. As a relatively low-resource assessment, SJTs are claimed to be a cost-efficient methodology compared with resource intensive assessments of non-cognitive attributes [5] like the MMI.

### Research context

The National Assessment Centre (NAC) model that provides the context for this research was organised and implemented by General Practice Education and Training Limited (GPET), which managed the Australian General Practice Training (AGPT) program (http://www.gpet.com.au) and is funded by the Australian Federal Government.

#### GP specialty training in Australia

Since 2000, GPET had overseen a regionalized system of general practice education, delivered through 17 regional training providers (RTPs) across Australia. RTPs deliver training towards two vocational endpoints recognised by Medicare Australia, which runs a publicly funded universal health care scheme. These are Fellowship; of the Royal Australian College of General Practitioners (FRACGP), and of the Australian College of Rural and Remote Medicine (FACRRM). The program consists of a three- or four-year full-time commitment, which may be reduced with recognition of prior learning. During training, registrars acquire practical experience in different training locations, including teaching hospitals, rural and urban practices, and specialised medical centres that provide health care for Aboriginal and Torres Strait Islander peoples, and people from socially disadvantaged groups. Registrars also acquire experience in extended skills/advanced specialised training, and can pursue other areas of relevant interest such as procedural general practice and academic posts. Training is conducted within accredited medical practices and hospitals and is supervised and assessed by accredited general practitioners. The training includes self-directed learning, regular face-to-face educational activities and in-practice education. Relevant college assessments are undertaken throughout or at the end of training to achieve fellowship and eligibility for specialist (general practitioner) registration.

#### AGPT national assessment process

The purpose of NAC was to determine who should qualify each year for the limited number of places available for Australian General Practice Training (AGPT) posts (n = 930 in 2011, and 1200 in 2012) in a transparent, fair, equitable way, based on both national and international best practice. The selection process needed to be consistently implemented in a highly diverse workforce training environment, which covers practice in urban; regional, rural and remote general practice. The aim of the NAC approach was to assess the candidates’ entry-level capability to perform as a GP registrar [27]. Invitation to attend an NAC was dependent on meeting eligibility criteria. In Australia these are complex (http://www.gpet.com.au), and relate to citizenship, medical qualification, medical registration and training program registration. For Australian qualified doctors, AGPT anticipates that specialty training will begin at the completion of postgraduate year two. There are, as in many countries, specific issues for international medical graduates (IMG), wishing to undertake postgraduate training. First, for medical registration, IMGs were required to have passed an Australian Medical Council competence test or demonstrate equivalence of their training. Secondly there has been a ten-year moratorium on IMGs post-Australian registration, requiring them to practice in areas of designated workforce need, mostly outside the major urban centres.

Following a pilot study in 2010, GPET introduced the NAC approach nationally in 2011, and was the first specialist professional organisation responsible for medical training in Australia to do so. In 2012, the selection process involved all 17 RTPs, which came together with GPET to run 11 NACs across Australia. Whilst attending one of the NACs in 2012, eligible candidates took a 6 station MMI and one of five versions of a written 50-item SJT. Those candidates with a satisfactory AGPT banding score (combined SJT and MMI scores) were passed to their preferred Regional Training Provider (RTP). RTPs took the candidate list and associated scores and matched candidates to supervisors in a locally determined way.

In the context of this national assessment approach into GP training, our research questions were:

– Can interviewers make reliable and valid decisions about the non-cognitive characteristics of candidates with the purpose of selecting them for entry into general practice training using the MMI?

– What is the concurrent validity of the MMI with the SJT?

## Methods

### Blueprinting procedures

This national selection process had been blueprinted with the facilitation of an external consultant against the expected competencies of entry-level registrars in the domains of practice defined by the relevant professional colleges (RACGP and ACRRM). These domains included communication, clinical skills, population health, professionalism, organisational areas and an assessment of personal attributes (including the capacity for self-reflection and awareness of the impact of cultural issues on delivery of primary health care) [1]. These domains were articulated into four distinct areas of assessment for the purpose of developing the MMI questions; Vocation/Motivation, Communication, Organisation/Personal Management, and Personal Attributes.

### Selection process organisation

Whilst attending one of the NACs in 2012, candidates sat a written SJT either before or after completing the observational MMI. The MMI circuit had six stations, each lasting 8 minutes, with a two-minute turnaround between stations. Candidates were required to read the MMI question before entering the room. Following completion of the mini-interview with a single interviewer, each candidate rotated through the circuit meeting a different single interviewer at each station. Between circuit iterations, interviewers moved to different stations, to avoid interviewer fatigue. Thereby over the course of several circuits, interviewers saw multiple candidates at each of two or more stations. Candidates were corralled on the interview day for test security reasons even though prior candidate knowledge of MMI scenarios would not be expected to make a significant impact on overall scores [28].

### MMI question development

**Figure 1:**
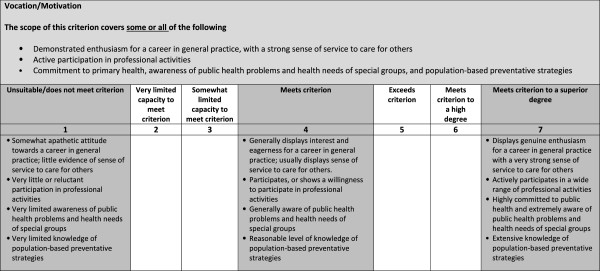
Guidance for interviewers for assigning marks in the MMI using a behaviourally anchored rating scale for a question of “Why do you want to be a GP”.

In the literature MMIs can be principally situational based [10,13], behavioral based [1] or contain a mixture of both types [29]. For this iteration of the NAC, all six MMI questions were of the behavioural type, and were created by an external organisational psychology consultant in a workshop with experienced GP supervisors. In correlating a *behavioural* MMI and a *situational* judgement test, it is worth drawing the theoretical distinction between the two types of questioning. The aim of behavioural questions is to collect evidence of past behaviour in the context of relevant job related situations. It is widely reported that the best predictor of past behavior is future behavior. An example behavioural MMI question might begin with an opening such as “Tell me about a time you had an angry patient. What was the situation? This might then be followed by prompts such as “What action did you take? “or “What was the outcome?” In contrast, situational interview questions [30] are based on the critical-incident technique, and typically begin “Imagine you are working in the emergency room at the weekend when you are asked to see an aggressive patient as an urgency?”In the NAC, MMI interviewers were provided with the MMI behavioural question, for example “Why do you want to be a GP?” The question and the interviewer prompts were matched to a behaviourally anchored rating scale (See Figure 1), which indicated the domain area of the question, the scope of the question, and the criteria and standards of anticipated interviewee performance. The prompt questions, were intended to promote a breadth of likely responses. Interviewers were encouraged to use in-depth probing questions to evidence the claims candidates had made about their past behaviours. Examples of good probing questions included “How did you tackle that situation?” and “How did you respond to those comments?”

### MMI marking schema

For each non-cognitive domain e.g. vocation/motivation, the marking schema included the scope of the criterion e.g. enthusiasm for a career in general practice, which was to be marked using a seven point rating scale. This ranged from 1 (unsuitable/does not meet criterion) to 4 (meets criterion) through to 7 (meets criterion to superior degree). For each anchor, descriptors were provided to indicate examples of the ways in which candidates might meet the criteria in the interview (see Figure 1).

### MMI interviewer training

Senior GP medical educators from each of the 17 RTPs undertook a train the trainer session, led by the external consultant. The objectives of the session were to familiarize interviewers with the assessment blueprint, how to ask behavioural questions, guidance for the use of behavioural probing questions, avoidance of common sources of interviewer subjectivity, and the use of the behaviourally anchored rating scale. During the workshop interviewers were encouraged to self-evaluate their confidence in using the behavioural MMI approach. These educators then ran similar workshops back in local RTPs for their local interviewers. All interviewers were provided with a detailed interviewer training guidebook.

### Situational judgement test development

The 50-item SJT broadly covered three domains: problem-solving and analytical skills; professional & ethical skills; and clinical performance and knowledge. Test items were generated in workshops with GP supervisors and medical educators within a specific blueprint (see Table 1). The answers to the SJT items were based on a concordance study panel made up of subject matter experts who had not been involved in the item development process. The final test format was typically made up of 32 ranking items (each with 5 response options) and 18 multiple response items (‘choose 3′, each with 8–10 response options). The total maximum score was 800. Because of test security issues arising over the testing period of 2 weeks, 5 parallel versions of the SJT were offered across the 11 NACs, which because of logistics varied from 44 to 50 questions. Candidate scores were adjusted to take account of the varying length of the tests.

**Table 1:** Blueprint for the three sections of the situational judgment test

**1. Analytical/problem solving:**	• Ability to investigate, analyse and synthesise complex information critically, to make rational evidence-based judgments and generate appropriate solutions.
	• Awareness of holistic aspects of patient care and how to manage their influence.
**2. Professional/ethical:**	• Demonstrated professional demeanour, shows respect for the views of others; Commitment to life-long learning and continuous professional development;
	• Acceptance of professional code of ethics and legal obligations such as special duty of care, equity of access, confidentiality requirements, honesty and integrity;
	• Commitment to maintain professional standards;
	• Ability to develop professional networks.
**3. Clinical knowledge:**	• Sufficient knowledge of how to manage common acute & chronic problems and how to recognise & respond to significantly ill patients within posed clinical problems;
	• Ability to develop working diagnoses;
	• Ability to judiciously prescribe medication and order investigations;
	• Ability to apply clinical knowledge effectively and appropriately

### Statistical analysis and decision-making procedures

Data were available from an AGPT held database, which recorded candidate demographics and assessment scores, and decision-making data. Interviewer data was available from an anonymous survey investigating acceptability of the NAC process, which is not reported here. Applying the method of Crossley et al. [31], we used Generalisability theory to provide dependable estimates in an unbalanced design, which was suitable for the naturalistic data we had available. The NAC was initially considered as a facet. Since it contributed less that 1% to the variance, it was withdrawn leaving a simpler model for further analysis. Candidates were fully crossed with MMI questions with each candidate attempting the same six MMI questions. Interviewers were partially crossed with questions (most interviewing at 2 or more out of 6 possible questions). Candidates were also partially crossed with interviewers with each candidate seeing 6 out of the total of all interviewers used (n = 254). We applied a random effects model specifying separately the effects due to ‘candidate’, ‘MMI question’, ‘interviewer’, ‘MMI question*interviewer’, ‘candidate*MMI question’ and ‘candidate*interviewer.’

The subsequent D-study modelled changes in reliability when different test formats were applied. We calculated the generalisability co-efficient G and standard error of measurement (SEM) from the variance estimates. We calculated the variance components of the MMI using the MINQUE procedure in SPSS 20. We re-analysed with ANOVA Sum of Squares Type III in order to report the degrees of freedom assuming the same level of effect sampling as in the MINQUE procedure [31]. We derived a confidence interval using the absolute standard error of measurement (SEM) [31] to explore the precision of ranking and decision-making. Pearson’s coefficient was used to assess the linear relationship between the MMI and the SJT and its subsections. We obtained an estimate of the relationship between our predictor (MMI) and criterion (SJT) variables under conditions where the magnitude of the correlation coefficient was not distorted by the unreliability in the predictor and criterion variables (attenuation) and restriction in the range of the predictor. In our situation although candidates were selected strictly on the basis of their combined MMI plus SJT scores we had the same population sample for MMI and SJT scores. Accordingly, correlations between the MMI and SJT were disattenuated for the unreliability of each of the measures, and not for the restriction of range [32].

#### Ethical considerations

The University Ethics committee approved the research. All candidates were reassured that data was strictly de-identified to protect participant privacy and reported in an aggregated manner (30).

## Results

Candidates were being selected for a total of 1200 training places. Data was available from 1382 candidates, 254 interviewers, six MMI questions, and 11 assessment centres. Of the 1382 candidates, 62.3% were female and 37.6% male. Age ranged from 22 to 65 years, with a mean age of 32.1 (standard deviation of 6.9), although the distribution was positively skewed. Candidates were born in 77 different countries with 588 candidates (42.5%) being Australian born. Candidates obtained their primary medical qualifications from 48 different countries, with 877 (63.5%), having obtained them from Australia. Of the 1382 candidates, 1061 (76.8%) candidates accepted an offer. with twenty-five (1.8%) declining the offer.

**Figure 2:**
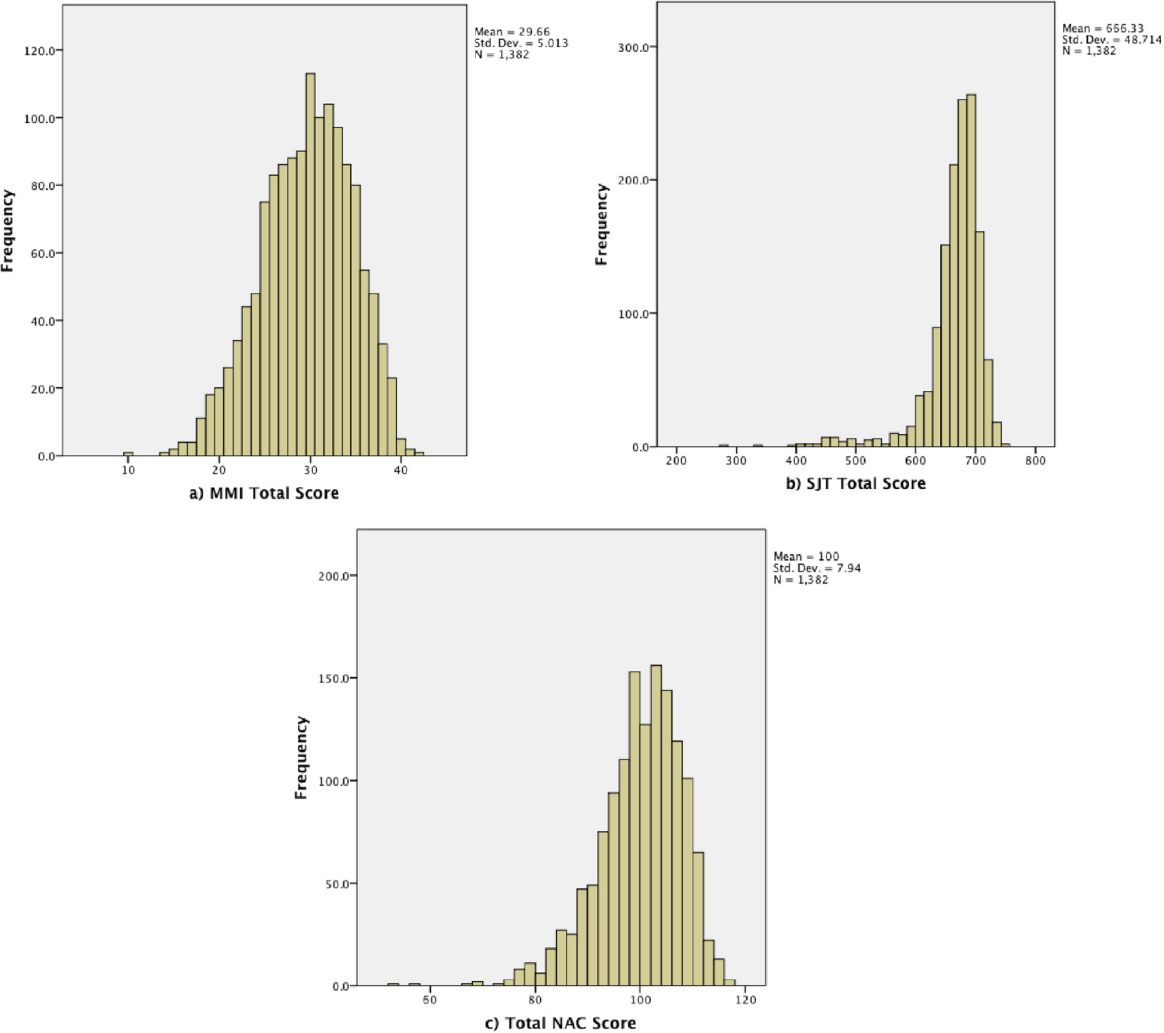
Histograms of the raw score distribution of; panel a) MMI Total Score (max = 46), panel b) SJT Total Score (max score = 800), and panel c) Total NAC Score (max= 120).

Of the 194/254 interviewers (76.4%) on whom we had data, 52% were female and 48% males, with nearly half (45%) aged between 51 and 65 years. Most interviewers (73.5%) had had some previous MMI interview experience. Most had a medical degree (87.6%) but there was some representation of other occupations, largely senior administrators. The number of candidates assessed at the individual assessment centres ranged from 22 (1.6%) to 303 (21.9%).Six MMI questions were used across all interviews and interviewers. The MMI total score was normally distributed, with a mean raw score of 29.7/42 (SD =5.0) or 70.6% (SD = 5.0%), with raw scores ranging from 10 to 42. (see Figure 2 panel a). The mean raw score of those who were not offered a place was 25.1/42 (59.8%) (SD = 4.7 [11.3%]).The SJT total scores showed a steep negative skew (see Figure 2 panel b), with most candidates scoring highly with a mean = 666.3 (82.4%) (SD = 48.7 [5.9%]). The minimum score was 285 (35.6%) and the maximum 755 (94.4%). The mean score of those not offered a place was 627.5 (76.4%) (SD = 67.6 [8.2%]).The NAC total assessment score used to determine the AGPT ranking band was calculated using a Z-score (Total Score-Mean Score/Standard Deviation) and a T-Score (Z-Score*10 + 100) for each assessment measure. For each candidate, the MMI and SJT each contributed 50% of the total mark ((SJT T-Score*0.5) + (MMI T-Score*0.5)), which made up the NAC score. These were normally distributed with scores ranging from 52.3 to 117.38 with a mean of 100 (SD = 7.9) (see Figure 2 panel c). The mean score of those not offered a place was 91.5 (SD = 7.9).

### MMI reliability

The variance components (Table 2) show that for one interviewer and a single MMI question, 28% of the variance between scores was due to candidate-to-candidate variation with the remaining 72% due to unwanted factors. This measurement error relates principally to interviewer subjectivity. This included the first order effect of interviewer stringency (9%), and the second order effect (i.e. interactions) of the varying views that interviewers had of a particular candidate, which accounted for 40% of the variance. Interviewer question-taste (3%) reflected one assessor marking generously on a particular question and stringently on a different one. The other significant error related to candidate question-aptitude, which reflected context specificity, the tendency for a candidate to perform well on one MMI question and poorly on another, and accounted for 18%.

**Table 2:** Variance components of MMI scores for partially crossed naturalistic data from a national assessment centre for selection into GP specialty training

**Component and their interaction**	**Explanation of interactions**	**Variance component estimate**	**Proportion of total variance from each factor (%)**	**Effect sample (degrees of freedom-df)**
Candidate	The consistent differences between candidates’ ability across interviewers and MMI stations	0.47	28%	1381
MMI question	The consistent differences in MMI station difficulty across candidates and interviewers	0.02	1%	5
Interviewer	The consistent differences in interviewer stringency across candidates and interviewers	0.15	9%	241
Interviewer with MMI question	The varying question-specific stringency of interviewers between MMI questions across candidates	0.06	3%	693
Candidate with MMI question	The varying MMI question-specific difficulty between candidates across interviewers	0.30	18%	6905
Candidate with interviewer	The varying views that interviewers have of candidates because of their differing perspectives	0.67	40%	6905

The G co-efficient of 0.70 was in the upper reported range for MMIs in the literature of 0.55-0.72 within a high stakes postgraduate setting [8] and on a reasonably large sample size (n = 1382). Following mathematical modelling in a Decision study, the generalisability co-efficient for a six question MMI was 0.7; to achieve 0.8 would require ten questions (see Table 3). The reliability of the SJT varied according to the five different versions of the test from 0.74 to 0.87, as reported in previous iterations elsewhere [5,26].

**Table 3:** Decision study modelling changes estimates of a +/- 95% confidence interval (=SEM × 1.96) around cut score (4/7) and reliability when increasing the numbers of MMI questions manned by a single interviewer

**No. of MMI stations**	**Estimate of SEM × 1.96 to provide +/- 95% confidence interval around cut score**	**G Coefficient**
4	1.07	0.61
6	0.88	0.70
8	0.76	0.76
10	0.68	0.80
12	0.62	0.82

### MMI validity

There was a modest raw score correlation (r = 0.26, n = 1382) between the two assessment measures. We assumed the MMI was the predictor variable and attenuated the correlation because of the difference in reliability between the SJT scores (mean r = 0.81) and the MMI (r = 0.7), [32] giving a disattenuated correlation of 0.35. An exploration of raw correlations between the three different subsections of the SJT with the MMI showed correlations of problem-solving and analytical skills (0.19), professional & ethical skills (0.18) and clinical performance and knowledge (0.24) suggesting the strongest relationship between the MMI and the SJT was in the area focussed on clinical knowledge.

### MMI decision making

**Figure 3:**
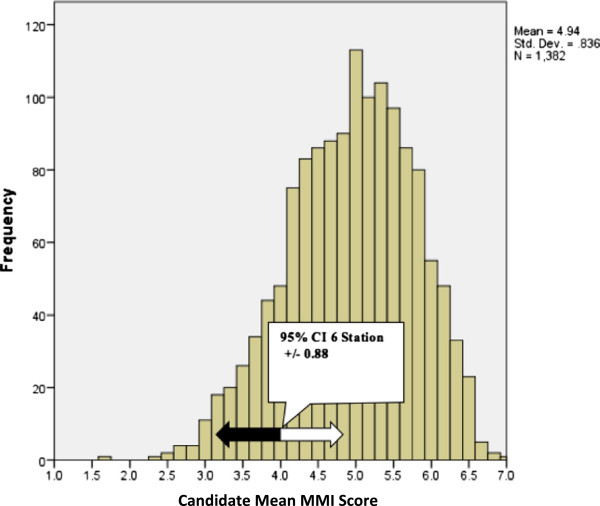
Histogram of candidates’ mean six-station MMI scores providing a +/- 95% confidence interval, which has been placed around the minimum satisfactory standard on the scale, 4/7.

Variance components from the G study were combined to provide a standard error of measurement (SEM) to create a 95% confidence interval around the candidates’ mean MMI total score (29). The SEM = √ error variance. The SEM was 0.92 giving a 95% confidence interval of +/- (1.96 × SEM) = 0.88. We put the 95% CI around the pass/fail score of 4/7 i.e. meets the criteria or is greater on the marking schema (see Figure 3). This would require candidates’ mean MMI scores to be greater than 3.12 (44.6%) and 23/1382 candidates (1.7%) failed this standard. Of interest is that those candidates who would have failed the MMI had SJT scores ranging from 62.8% to 87% with an average of 77.4%. The mean SJT score of those not offered a place was 76.4%. Adjusted NAC scores ranged from 69.9 to 90.3 with an average of 84.0. Given that the pass score for the SJT had not been determined, we were unable to apply an agreed decision making process to the adjusted NAC scores.

## Discussion

We have reported the results of a high stakes National Assessment Centre process to determine entry into a specialist (general practitioner) training program in which, for the first time, a combination of an observed Multiple-Mini-Interview (MMI) and a written Situational Judgement Test (SJT) was used. In our study, the MMI was observational, focussed on non-cognitive skills, and used behavioural type questions. The written SJT was also focused principally on non-cognitive skills, and used situational type questions. In relation to the construct validity of the MMI in postgraduate settings, we have demonstrated that interviewers can make moderately reliable and valid decisions about the non-cognitive characteristics of candidates with the purpose of selecting them for entry into general practice training using the MMI. Our data confirms that as in other MMI settings, the main source of error is interviewer subjectivity [13,33,34], as opposed to context specificity, which is often a major source of bias in communication OSCEs [35,36]. We also demonstrated for the first time a relationship between the MMI and the SJT. We discuss these findings in more detail.

### MMI reliability

The finding that a significant proportion of variance (28%) is related to the desired behaviours of a candidate resonates with Dore et al’s study, which included a significantly smaller sample size. [8] In examining the sources of interviewer subjectivity, a number of conclusions can be drawn. The highest source of variance related to views that interviewers have for particular candidates, because of their particular perspective or pre-conception, which accounted for nearly half (40%) of all the variance. This represents a large discrepancy between a candidate’s scores as a result of individual interviewer bias – or the snap judgments regarding a candidate that interviewers make. In our study there was a higher proportion of candidate variance compared for example with the 22% in a graduate entry situational style MMI question. [13] This might reflect the greater certainty amongst interviewers in determining the trainability of a doctor as opposed to determining the aptitude of a student for undertaking a medical degree. Equally it might reflect the lack of independence between the different MMI questions, i.e. they were testing very similar things. Addressing interviewer training and tightening definitions in the marking criteria has traditionally been used to address interviewer subjectivity, particularly in situations where the interviewer pool, in our case GP supervisors is finite and can’t be further diversified. However, neither strategy alone or in combination, has resolved the persistent challenges of interviewer variability, and there is a need for novel evidence-based approaches, which have so far been discussed in the context of work-based assessment around rater cognition [37-39]. Interviewers may have used different schemas in judging candidate performance, in a process that has similarities to clinical reasoning, the notion of making some instant and intuitive decisions about candidates based on pattern recognition and making more considered and analytical decisions [38]. By investigating the perceptual and processing capacities of our interviewers, and the schema they operate by, and then aligning the scoring system, we may be able demonstrate improved discrimination between candidates in future iterations of the MMI.

Although interviewer stringency leniency accounted for 9% of the variance in our study, it is generally thought that this is a relatively stable characteristic of interviewers, and is not impacted upon by training [40]. However, consideration could be given to adjusting candidates’ scores by using a measurement model [40], which accounts for the stringency/leniency of whichever interviewers the candidate saw. Increasing the number of MMI questions is another way in which reliability may be added to the MMI, particularly as a comprehensive question bank is developed [13], but can be problematic logistically. Our D study suggests that in order to achieve a reliability of 0.80, there would need to be 10 MMI stations, which was logistically impossible because candidates are required to sit the SJT on the same day. Although a minimum 6-station MMI with a G of 0.70 is recommended to ensure a balance between reasonable reliability and resources available, future flexibility in offering more MMIs might be afforded by developing on-line testing facilities for the SJT.

### MMI validity

The assessment blueprint guiding the content areas for the MMI and SJT had content validity because they was developed fit for purpose by organisational psychologists specialising in selection focussed assessment. However they were developed differently across two different formats, and the professional colleges (RACGP and ACRRM) would be advised to revisit the blueprints focussing on the anticipated attributes of GP registrars. Expected relationships of the MMI with independent external variables, such as the SJT provide some evidence to support the validity of its use in postgraduate settings [41]. There is also a pragmatic interest in the relationship, as it has been claimed that SJTs would be a more cost-efficient methodology compared with more resource intensive assessments of non-cognitive attributes, such as the MMI [5] The finding of a modest disattenuated correlation (r = 0.35) between the behavioural MMI and the SJT suggests that the two formats are testing differing non-cognitive aspects and should be retained on the argument of divergent validity. One advantage of situational questions is that all interviewees respond to the same hypothetical situation rather than describe experiences unique to them from their past. Another advantage is that situational questions allow respondents who have had no direct job experience relevant to a particular question to provide a hypothetical response. Where feasibility and cost constrain the number of assessment formats that can be used, it raises the question as to which best predicts GP registrar performance either in-training or in professional college examinations. This NAC principally focussed on non-cognitive characteristics of candidates. There has been international interest in postgraduate settings, to offer some testing of clinical competence, particularly where many candidates have received their medical degrees and early training in multiple settings, some of which are of varying quality. For example within the UK, the postgraduate selection community has favoured the combination of a cognitive test, the clinical problem-solving test (CPST) [6] with the non-cognitive SJT to ensure a broader coverage of desired candidate attributes. To date, in Australia, candidates’ clinical competence are assumed as being represented in either an Australian primary medical degree or passing an Australian Medical Council Accreditation Examination for international medical graduates. Perhaps, because of a lack of assessment in the intern and resident years, there has been sufficient concern about the clinical competence of borderline candidates that sections of the GP selection community have pushed for an element of clinical competence testing, alongside the SJT and the MMI. The relationship between the MMI and the clinical knowledge section of the SJT is of interest in this context. Decisions to determine the best combination of selection formats will likely require validity studies of the success of the MMI and SJT, individually and in combination in determining what best predicts observed performance in practice. Further debate is required amongst stakeholders to ensure that the validity of the MMI continues to have relevance when considering logistically sustainable combined measures of the trainability of entrants into specialist training.

### NAC decision making

Developing a cut score for the combined NAC score that is both psychometrically robust and acceptable to all stakeholders is a complex process. However, it is important to provide data on which to base these on-going discussions. Although no formal standard setting procedure was used in the NAC, we modelled possible standard setting procedures for future iterations. We had anticipated the MMIs reported precision [10,13] would allow relative ranking of candidates. From Figure 3, the MMI contains enough precision to suggest concern that 23 (1.7%) candidates had failed the MMI with 95% confidence. However, the confidence interval crosses three quartiles giving less than 95% confidence that a candidate at the bottom of the top quartile might behave better than a candidate at the top of the 3^rd^ quartile [31]. There needs to be acknowledgement that large-scale performance-based assessments are logistically complex and costly to run. Scores based solely on performance-based stations, such as the MMI require extended testing time to achieve acceptable generalizability, to which would be added time for question development and training. Combining scores from performance-based formats and written formats may improve test generalizability, and methods to do this already exist [42,43]. It could be possible that the combined NAC score was more generalizable than either of the two measures individually, and potentially a better use of resources. In considering the construct validity, generalizability and the precision of the combination of the MMI and SJT, more data would need to be made available on the detailed scoring of the SJT, in order for an acceptable methodology that all stakeholders had confidence in. Additionally, a method for providing a cut score for the SJT, for example with a modified Angoff, would need to be provided.

### Limitations of the study

The strength of this study was that it evaluated a high stakes National Assessment Centre approach, with sufficient numbers to ensure adequate sampling of all the factors. However, the study was a secondary analysis of a process that was conducted naturalistically and was constrained by what was logistically possible. As is often the case in such settings, there was no fully formalized design that assigned specific interviewers or a specific set of items to each MMI circuit, nor which version of the SJT they sat. We had initially anticipated that ‘candidate*interviewer’ and ‘candidate*MMI question’ interactions would be confounded [18] and included in the error term, because of the single interviewer within station design. However given the GLM procedure was able to provide estimates because there were enough degrees of freedom for this to happen. We therefore assumed a partially crossed model of generalisability to best reflect this particular setting [31]. In this study we were unable to link interviewer demographics and provide additional analysis about the impact of rater characteristics on interviewer subjectivity as we have done in previous studies [40].

## Conclusion

In a high stakes national assessment centre approach to selection into postgraduate training, we confirmed a behavioural MMI is a moderately reliable method of assessment. For the MMI, the largest source of identifiable measurement error related to aspects of interviewer subjectivity, suggesting further training of interviewers would be beneficial. We added to understandings of the construct validity of a behaviourally orientated MMI by showing a modest positive correlation with situationally orientated SJT scores, with the most significant element relating to the clinical knowledge subdomain of the SJT. We demonstrated a small proportion of candidates who would have failed the MMI. In order to justify long term sustainable use of the MMI in a postgraduate assessment centre approach, more theoretical work is required to understand how written and performance based tests of non-cognitive attributes can be combined, in a way that achieves acceptable construct validity, generalizability, and reasonably precise decision making processes. Stakeholders need to have confidence that the combined measure is of value in measuring trainability of trainees and registrars in a way that is logistically sustainable. Predictive validity studies are required to determine to what extent the MMI and the SJT both singly and in combination predict both in-training program performance and professional college membership examination scores.

## Competing interests

Over the past three years CR and his team, TC, AB, MF, and KM have held a contract to evaluate the MMI element of the NAC process described in this paper. Additionally CR has received payment for workshops about selection processes in general for professional colleges. MG was the Manager Selection at GPET at the time of this study.

## Authors’ contributions

CR conceived of the research question, conducted the literature review, data analysis and interpretation, in particular the generalizability studies, and wrote the first draft. TC managed the data collection, data analysis, interpretation and initial report writing. MF, AB, MG and KM, were all involved in data collection, data interpretation, critical review of manuscript development, and approved the final version. All authors read and approved the final manuscript.

## Pre-publication history

The pre-publication history for this paper can be accessed here:

http://www.biomedcentral.com/1472-6920/14/169/prepub
